# Perseveration on cognitive strategies

**DOI:** 10.3758/s13421-023-01475-7

**Published:** 2023-10-24

**Authors:** Patrick P. Weis, Wilfried Kunde

**Affiliations:** Department of Psychology, Julius-Maximilians-Universität Würzburg, Lehrstuhl für Psychologie III, Röntgenring 11, D-97070 Würzburg, Germany

**Keywords:** Perseveration, Cognitive strategy selection, Cognitive offloading

## Abstract

To acquire and process information, performers can frequently rely on both internal and extended cognitive strategies. However, after becoming acquainted with two strategies, performers in previous studies exhibited a pronounced behavioral preference for just one strategy, which we refer to as *perseveration*. What is the origin of such perseveration? Previous research suggests that a prime reason for cognitive strategy choice is performance: Perseveration could reflect the preference for a superior strategy as determined by accurately monitoring each strategy’s performance. However, following our preregistered hypotheses, we conjectured that perseveration persisted even if the available strategies featured similar performances. Such persisting perseveration could be reasonable if costs related to decision making, performance monitoring, and strategy switching would be additionally taken into account on top of isolated strategy performances. Here, we used a calibration procedure to equalize performances of strategies as far as possible and tested whether perseveration persisted. In Experiment 1, performance adjustment of strategies succeeded in equating accuracy but not speed. Many participants perseverated on the faster strategy. In Experiment 2, calibration succeeded regarding both accuracy and speed. No substantial perseveration was detected, and residual perseveration was conceivably related to metacognitive performance evaluations. We conclude that perseveration on cognitive strategies is frequently rooted in performance: Performers willingly use multiple strategies for the same task if performance differences appear sufficiently small. Surprisingly, other possible reasons for perseveration like effort or switch cost avoidance, mental challenge seeking, satisficing, or episodic retrieval of previous stimulus-strategy-bindings, were less relevant in the present study.

**Supplementary Information:**

The online version contains supplementary material available at 10.3758/s13421-023-01475-7.

## Introduction

**Fig. 1:**
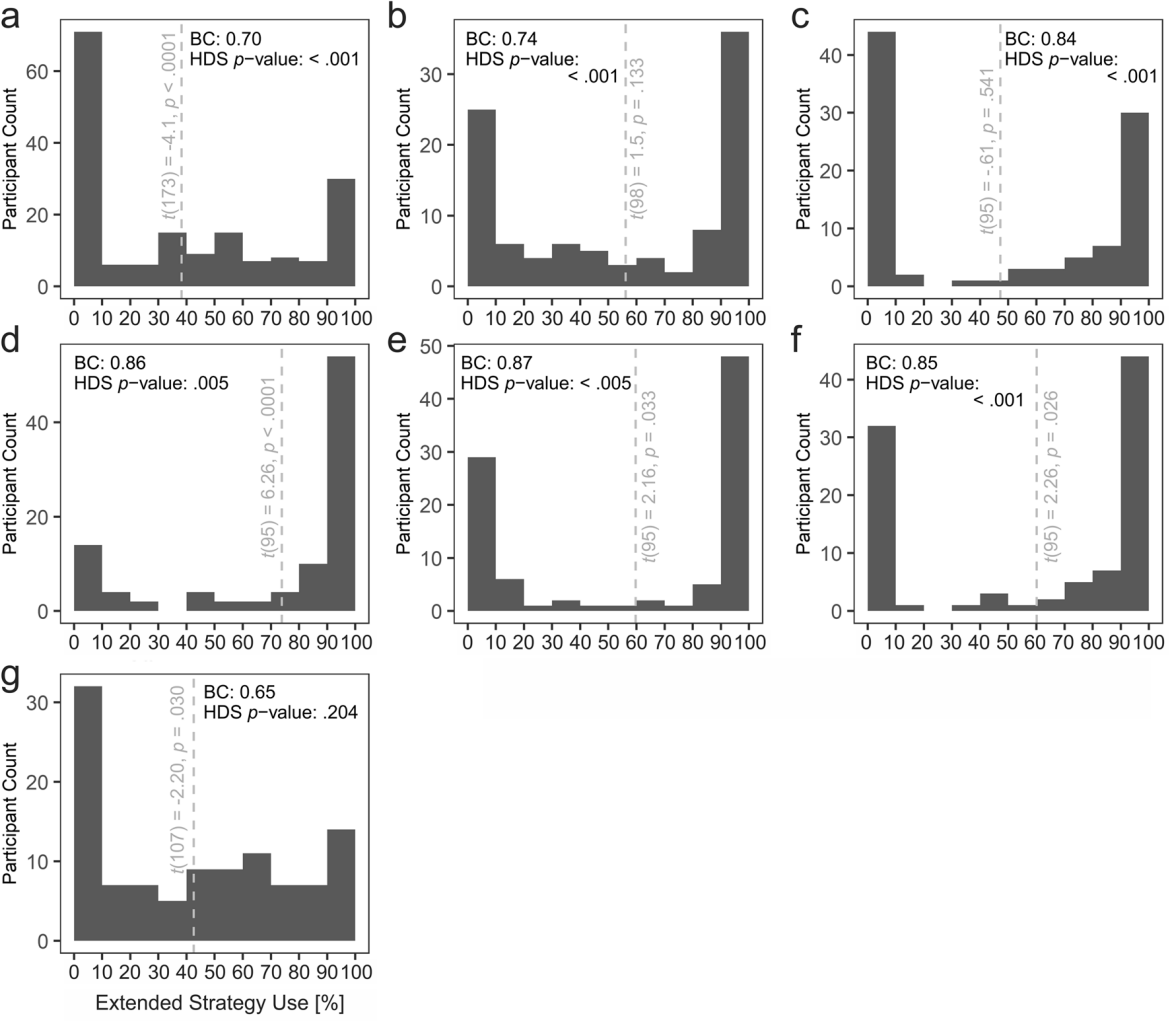
Perseveration in previous studies with a choice between an internal and an extended cognitive strategy. *Note*. In a multitude of different experiments with different paradigms, participants strongly preferred to use either an internal cognitive strategy or an extended cognitive strategy when given the choice between the two; a–g. Participants rarely showed weak or no preferences for one strategy. Perseveration is reflected by bimodal distributions in the present figures. Data was obtained from joint arithmetic and social problem solving with a telepresent agent (a; Weis & Herbert, 2022), from solving alphanumeric problems with or without help from a computer (b; Weis & Wiese, 2019a), from intention offloading with or without spatial manipulation on a computer screen (c–f; Scarampi & Gilbert, 2020), and from an object rotation task with mental rotation or manual computer-mediated rotation (g; Weis & Kunde, 2022). Dashed gray lines represent means with the *p*-value of one-sample *t*-tests testing against μ = 50% attached. Based on a .05 alpha level, a *t*-test *p*-value below .05 would suggest that one strategy was preferred over the other. A bimodality coefficient (BC) above 0.55 as well as a Hartigan’s dip statistic (HDS) *p*-value below .05 are commonly interpreted as evidence for a bimodal distribution

Past research investigating the choice between purely internal and partly environment-based cognitive strategies has shown that a large proportion of human problem solvers establish extreme behavioral preferences for one specific strategy (Fig. 1). What are the reasons for such *perseveration*? In the present study, we investigated whether perseveration reflects preference for superior strategies as revealed by accurate performance monitoring (see also Gray et al., 2006). If that was the case, we argue, perseveration should cease if performance of different strategies is equalized. Alternatively, if perseveration tendencies prevail despite comparable performance, reasons related to constraints of our minds would remain as possible reasons for perseveration. Such reasons would include reduction of the costs for deciding between strategies (Simon, 1956), of the costs for preparing and realizing strategy switches (Kiesel et al., 2010; Weis & Kunde, 2022), or potential benefits of omitting performance monitoring (Weis & Kunde, 2023).

## ‬Perseveration: Problem solvers rarely mix internal and extended cognitive strategies

Everyday problem solving relies on suitable cognitive strategies. For example, estimating the combined costs of buying a US$8 olive oil and US$3 of carrots can be done by slowly and sequentially counting upwards from eight or – if some arithmetic experience has been acquired – by quickly retrieving the correct solution from memory (analogously to algorithmic and retrieval strategies in Compton & Logan, 1991). In addition to such *internal* cognitive strategies (referred to as internal strategies), human problem solvers can also rely on a plethora of cognitive strategies that are *extended*, i.e., go beyond the brain and include some physical means, be it the own body or an external device (referred to as extended strategies)*.* For example, one could use fingers in addition to working memory to support counting or entirely omit counting and use a smartphone-based calculator instead. Note that in the present article, we use the term *extended strategy* instead of *external strategy* because some internal processing is involved in any environment-based information processing as well. For a more nuanced discussion of internal strategies, we refer the reader to Compton and Logan (1991), and of extended strategies to Clark (2011).

It is evident that deciding between strategies is a ubiquitous and relevant endeavor in today’s cognitively complex and computerized environments. For example, favoring a shopping-list-strategy over a memory strategy will likely improve shopping performance (Risko & Dunn, 2015). But similarly, mixing both computer-based and memory-based strategies can reduce memory-based inference and thus boost memory performance (Storm & Stone, 2015). More generally, using extended strategies can have the potential to free mental resources (e.g., Ballard et al., 1997), but also induce cognitive biases in which computer-based help is misinterpreted as own intellectual achievement (Fisher et al., 2015), and so on.

These potential consequences of strategy choice exemplify the relevance of a previously observed intriguing behavioral pattern: When explicitly instructed to choose between an internal and an extended strategy, human problem solvers seem to frequently stick to just one of them. In other words, they perseverate. Thus, with two strategies available, rather than employing both about 50% of the time, one is chosen much more often than the respective other. Group-wise, this leads to a bimodal distribution with peaks at low *and* high extended strategy use or a unimodal distribution with peaks at low *or* high extended strategy use; see Fig. 1.

Such bimodal strategy use has been found in situations with no prior experience with either strategy, which indicates some sort of reluctance to explore performance of the respective other strategy (Fig. 1a; Weis & Herbert, 2022). However, even if participants had extensive experience *only* with an internal strategy, strong perseveration was found (Fig. 1b; Weis & Wiese, 2019a). Interestingly, perseveration was of a similar size, both for the familiar internal strategy and for the unfamiliar extended strategy, suggesting no inclination to stick with the well-known strategy. Similar results have been found in a study that also featured previous experience only with the internal strategy (Fig. 1c; Scarampi & Gilbert, 2020). Complementarily, other experiments from that study with independent samples also featured previous experience only with the extended strategy (Fig. 1d) or mixed previous experience (ten trials internal and one trial extended in Fig. 1e; one trial internal and ten trails extended in Fig. 1f). Even with such widely varying previous exposure to both types of strategy, perseveration on both internal and extended strategies was apparent in all three experiments.^1^ Lastly, perseveration was also found after an extensive practice of 144 trials for each internal and extended strategy (Fig. 1g; Weis & Kunde, 2022) – and, interestingly, perseveration was found^2^ in this study even though participants were instructed to, and actually did, switch frequently – in about 50% – of these practice trials. Taken together, in the experiments depicted in Fig. 1, 54% of participants chose one strategy in more than 90% of trials (22% internal, 32% extended). Perseveration was also apparent with more conservative criterions: 45% of participants used one strategy in more than 95% of trials (18% internal, 27% extended) and 32% of participants exclusively used one strategy without switching at all (14% internal, 18% extended). Note that the influence of performance on perseveration in these studies is hard to determine post hoc since no robust and unbiased performance estimates of both strategies had been acquired, which we aim to remediate in the present study. However, also note that the influence of performance on strategy choice without perseveration has been consistently shown (e.g., Gray et al., 2006; Gilbert, 2015; Walsh & Anderson, 2009; Weis & Wiese, 2019b).

Apart from studies with both internal and extended strategies, intra-individual preference for one specific strategy has also been observed in a study comparing two internal strategies. Specifically, it was shown that a large proportion of participants (~one-third) persisted on using an explicit rule to solve a categorization task rather than gradually switching to “automatic” memory retrieval (Bourne et al., 2010). To what extent the choice between two internal strategies is similar to the choice between an internal and extended strategy is an intriguing question that, however, is beyond the scope of the current article. To reiterate, in the present work, we aim to further investigate the phenomenon of cognitive strategy perseveration in settings with a choice between an internal and an extended strategy.

## Stimulus rotation as a testbed for choosing between internal and extended cognitive strategies

The omnipresence of smartphones, laptops, and regular paper-based notebooks provides a continuous supply of extended cognitive strategies. This omnipresence renders extended strategies similarly available to internal strategies in many everyday situations. In the present study, we created an analogue setting with an availability of both types of strategies. Specifically, we asked participants to solve an object comparison task with either manual or mental rotation. Manual rotation (Wohlschläger & Wohlschläger, 1998) is an extended strategy in which problem solvers rely on keyboard buttons or knobs that result in the rotation of an object on a screen. Analogously, mental rotation (Shepard & Metzler, 1971) is an internal strategy in which the rotation process is taking place via mental imagery (Kosslyn et al., 2001). Both mental and manual rotation can be used to infer what turning an object would look like.

Interestingly, both rotation strategies rely on partially overlapping cognitive resources. Specifically, it was shown that hand movements within the rotation plane interfered with the mental rotation process, which strongly suggests that mental rotation is not independent of manual rotation (Wohlschläger & Wohlschläger, 1998). In fact, this first study on manual rotation even found nearly identical response times (RTs) for mental and manual strategies for two-dimensional^3^ object rotation. However, this striking similarity between mental and manual rotation regarding performance could not be confirmed in another study using two-dimensional object rotations. Specifically, both accuracy and RT differences have been found (Weis & Wiese, 2019b). Furthermore, it was shown that participants in a free choice between a manual and mental rotation condition preferred the strategy that would better fit a participant’s current performance goal (i.e., maximizing accuracy or speed). Whereas these latter findings certainly do not contradict the idea that shared mental processing is necessary for both mental and manual rotation, they do also indicate the existence of divergent processing, which can lead to divergent performance. Yet, such divergent performances between internal and extended strategies are undesirable in the present study for reasons explained in the next paragraph, which necessitates a calibration procedure to adjust for possible differences.

### Current study

#### Individual performance differences as source of perseveration?

Why do human problem solvers frequently perseverate on one specific cognitive strategy to solve a task? Here, we investigated whether perseveration is caused by differences in individual performance profiles. If a participant consistently^4^ performs better with an internal than with an extended strategy, it would be reasonable to perseverate on the internal strategy. To explain the often observed bimodal distribution of strategy use, this hypothesis would suggest that interindividual performance differences between strategies exist and that the extent of performance differences predicts the degree of strategy perseveration. Our approach to test this proposal in the present study was to calibrate performance as well as possible, such that each individual participant performed comparably well with both strategies. If participants still exhibited perseveration even after calibration, this would clearly speak for reasons beyond accurate performance monitoring, such as satisficing (Simon, 1956), avoiding switch costs (Dunn & Risko, 2019; Kool et al., 2010; Schillemans et al., 2012; Weis & Kunde, 2022), or avoiding mental load that comes with internal cognitive strategies (e.g., Ballard et al., 1997; Sachdeva & Gilbert, 2020). This study was purposefully designed to target such performance-independent reasons for perseveration. If considerable perseveration persisted even after controlling for performance, the logical next step would be to disentangle the contributions of the alternative reasons just mentioned.

##### Hypotheses

Experiment 1 was preregistered (https://osf.io/4pzh7). We thus describe the hypotheses as stated before data collection (though in a slightly different order for improved readability). Our first two hypotheses concerned the quality of our performance adjustment procedure. If our calibration procedure was successful, not only should mean performance of strategies be comparable at the end of calibration (*H2*) regarding both response times (*H2a*) and accuracy (*H2b*), but any remaining performance differences between strategies should be uniformly or unimodally rather than bimodally distributed (*H1*) again regarding both RTs (*H1a*) and accuracy (*H1b*). This would rule out the possibility that any bimodal distribution of strategy choice occurring in a choice block after calibration was caused by a bimodal performance distribution. *H3* is the main hypothesis regarding choice behavior. *H3* posits that strategy perseveration occurs despite performances of the available cognitive strategies being highly similar. Under these circumstances, perseveration might similarly occur with internal and extended strategies (*H3a*), or, as previous research suggests (e.g., Gilbert et al., 2019; Touron, 2015), be more likely to occur with extended strategies (*H3b*). If confirmed, this would indicate that performance differences are not the main reason for perseveration behavior and open the door for further investigations regarding performance-independent reasons.

## Experiment 1

### Methods

#### Participants

Based on the power calculations detailed below, data collection stopped after 54 participants (age mean 26.9 years, range 19–54 years; 37 female, 17 male) had been measured after participant exclusions. Sixteen participants were excluded based on preregistered criteria: Seven participants did not rotate in at least 85%^5^ of all extended blocks combined (i.e., blocks 2, 4, 6, and 8). This criterion was necessary to ensure that participants did not use the internal strategy during extended blocks or, in other words, to ensure that the performance estimates for the extended strategy were valid. Two participants performed outside of 2.5 standard deviations around the group RT mean, and five participants performed below 80% accuracy in all internal blocks (i.e., blocks 1, 3, 5, 7, and 9). Similar criteria were used to an earlier study with the same paradigm (Weis & Wiese, 2019b) to ensure reasonable data quality. Additionally, one participant was excluded because accuracy calibration (15% accuracy calibration difference between internal and extended strategy) and one because RT calibration (2.7-s calibration difference between internal and extended strategy) was substantially worse than for all other participants.

The power calculations were based on what we expected to be the least powerful test: whether calibrated performances of internal and extended trials in Blocks 7 and 8 are comparable (*H2*). Power estimations were made in R (version 4.1.1; R Core Team, 2013) with the *BESTpower* function from the BEST package (version 0.5.3; Kruschke, 2013). The power estimation was based on a target power of 0.9 with a 95% highest density interval within the ROPE from -200 to 200 ms, an estimated sample mean of 0 ms, and an estimated sample standard deviation of Δ(RT_internal_ – RT_extended_) of 300 ms. The resulting sample size of 54 also afforded sufficient power (> .9) for a ROPE from -2% to 2% accuracy based on an estimated sample mean of 0% and an estimated sample standard deviation of 2%.

After data collection, results indicated that we underestimated standard deviations. Actual standard deviations were 687 ms and 3.6%, respectively. Re-running power estimations with updated standard deviations resulted in a power of the accuracy-based ROPE analysis of about 0.85 and of the RT-based ROPE analysis of only about 0.2. If willing to broaden the RT ROPE from -200 to 200 ms to -350 to 350 ms, power would increase to about 0.85 again. We decided to stick with the pre-registered sample size while keeping possible power issues in mind for the interpretation of results.

#### Apparatus

The study was presented at a distance of about 75 cm on an BENQ XL2411P 24-in. monitor set to a resolution of 1,920 × 1,080 pixels with a refresh rate of 100 Hz using MATLAB version R2016a (The Mathworks, Inc., Natick, MA, USA) and the Psychophysics Toolbox (Brainard, 1997; Pelli, 1997). Responses were recorded using a USB-connected standard keyboard and mouse. The stimulus rotation (see section Methods*: Task*) was updated at a frequency of about 50 Hz.

#### Stimuli

**Fig. 2.**
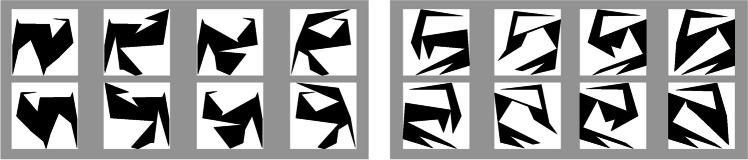
Example stimuli. *Note*. Each box shows the four unmirrored (top row) and mirrored (bottom row) stimuli at zero degrees belonging to one family for two (left and right box, respectively) example families. Note that only base but not working stimuli (see Figure 3a) were presented at zero degrees angle

A total of 48 black stimuli with 16 edges was created based on a procedure described by Attneave and Amoult (1956) and realized using a Matlab-based script provided by Collin and McMullen (2002); see Fig. 2. Size was adjusted so that each stimulus barely fitted in a square with a side length of 7.5 cm or, given that participants were seated about 70 cm in front of the screen, about 6.1° visual angle. All stimuli were either presented at zero degrees angle as created by the script or rotated before presentation, depending on the condition.

**Fig. 3:**
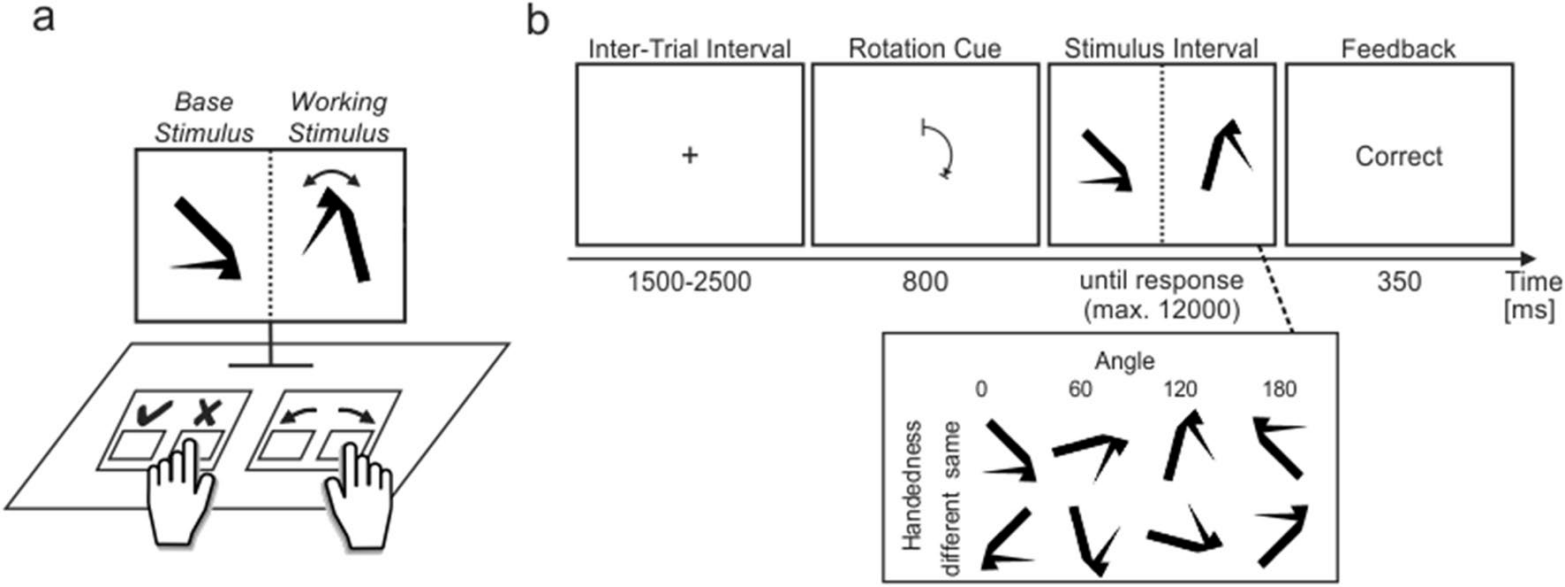
Extended rotation paradigm. *Note*. Participants had to compare the handedness of two stimuli that differed in their angular orientation and decide whether the left is the “same” (only rotated in two-dimensional plane) or a “different” (first mirrored, then rotated in two-dimensional plane) stimulus by pressing the appropriate key (✓ or ✗, respectively; the “a” key was labelled ✓, the “s” key was labelled ✗; **a**). For each trial with a specific shape, the base stimulus stayed identical whereas the working stimulus was altered using a handedness and angle transformation; **b**. To help their decision, participants were able to offload their mental rotation process onto a physical interaction with the keyboard that afforded rotating the working stimulus on screen (manual rotation). The coupling between rotation keys and working stimulus can replace a mental rotation process and thus constituted an *extended cognitive strategy*, in contrast to the mental *internal cognitive strategy*. Preceding each stimulus interval, a rotation cue indicated the preferred rotation direction and how long participants were advised to rotate. The rotation cue was used to make rotation across participants more comparable. During each stimulus interval, the base stimulus was then presented in the left and the working stimulus in the right half of the screen for 12 s or until a response was given. The trial terminated with feedback indicating whether the given answer was correct. The paradigm was adjusted based on a similar paradigm used in previous research (Weis & Wiese, 2018, 2019b, c)

Our study necessitated a sufficiently precise estimation of the Angle-RT slope parameter to warrant acceptable calibration of the extended strategy’s rotation speed. A higher slope parameter likely increases precision of the estimation by decreasing the relevance of RT noise. It is known that a high similarity between complex Attneave/Amoult stimuli is associated with a high Angle-RT slope (Folk & Luce, 1987). We therefore decided to use complex stimuli with many edges (i.e., 16). We therefore also decided to use similar stimuli: All stimuli had the same number of edges and every four stimuli were belonging to what is called the same shape *family*. Family members were created by distorting a prototypical shape using a family resemblance parameter (here: 0.1; for details, consult Collin & McMullen, 2002).

#### Procedure and task

**Fig. 4:**
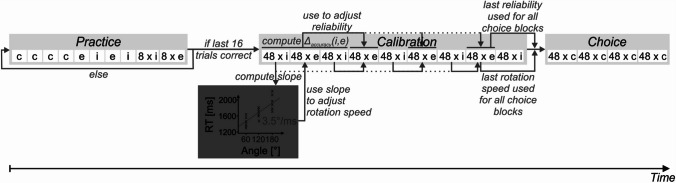
Procedure for rotation task trials. *Note*. Participants engaged in a practice, calibration, and choice section. Only if the last 16 trials of the practice section were answered correctly were participants allowed to advance to the calibration section. The calibration section was used to adjust performance parameters of manual rotation to match performance of mental rotation. Calibration started and ended with an internal block. The initial internal block afforded calibration of manual rotation during the first extended block. The terminal internal block was chosen to maximize participants’ use of the mental rotation throughout the choice trials (for a similar procedure, see Scarampi & Gilbert, 2020). Because participants are known to prefer manual over mental rotation in the present paradigm (Weis & Wiese, 2019b, c), the terminal internal block was incorporated to counter this preference and incentivize participants to use the mental rotation more frequently. The underlying rationale was that a more balanced use of mental and manual rotation on a group level allows for a better analysis of possible perseveration tendencies on an individual level (compare H3-1). *c*: choice trial, *e*: extended trial, *i*: internal trial

Participants were invited to the lab and asked to engage in the extended rotation paradigm (Fig. 3). Following instructions that asked participants to work thoroughly and avoid errors whenever possible, participants engaged in at least 24 practice trials. First, participants were instructed to use any of both strategies, then they were explicitly asked to use either mental or manual rotation, depending on the trial (compare Fig. 4). Practice trials had to be repeated if an error occurred during the final 16 practice trials. After successfully completing the practice section, participants engaged in a calibration section consisting of several internal blocks with mental rotation and extended blocks with manual rotation. Specifically, during internal blocks, mental rotation performance was measured and keyboard-based rotation was deactivated. In extended blocks, manual rotation parameters were then adjusted based on internal performance on an individual basis by (a) altering the speed at which the object rotated on screen and (b) adjusting the reliability of manual rotation. In other words, if a participant was slow at rotating an object using mental rotation, this procedure ensured that manual will also be slow. Specifically, the slope of a linear regression (independent variable: Angle, dependent variable: RT) was computed at the end of each internal block based on the RTs of all correctly answered trials in internal blocks so far. This slope was then used to adjust the rotation speed of manual rotation to mimic mental rotation. For example, if the slope of mental rotation was 2 (rotating for 1° needs 2 ms), the manual rotation (which updates every 20 ms) would rotate a stimulus by 10° every 20 ms while the rotation key was pressed. Similarly, if a participant committed errors using mental rotation, manual rotation was accordingly made less reliable (i.e., instead of rotating the working stimulus (see Fig. 3), the working stimulus in an *unreliable trial* would vanish after the rotation key was pressed and a text would appear indicating that “loading the rotated image failed”). Specifically, the difference in errors between the internal and extended block *preceding* another extended block is calculated. For each error participants committed more in the internal than in extended preceding blocks, two unreliable external trials were added to the respective extended block. For example, if a participant answered 45 out of 48 trials correctly in Block 6 (extended) and 44 out of 48 trials correctly in Block 7 (internal), two more unreliable trials would be added in Block 8 (extended). If there already were two unreliable trials present in Block 6, there would now be a total of four unreliable trials in Block 8. Two instead of one trial were added to accommodate for the 50% chance level. For the first accuracy calibration in Block 4, in addition to the mean accuracy of Block 2, the mean accuracies of both preceding internal Blocks 1 and 3 were used instead of only Block 3. Importantly, both reliable and unreliable trials were used for computing a participant’s extended strategy performance.

After completing the calibration section, participants were able to freely choose between mental and manual rotation in a final choice section. In fact, we reminded them at the beginning and after every 48 trials that they can freely choose between both strategies and that they should avoid errors as far as possible.^6^ If calibration was successful, any remaining perseverance in the choice section would be independent from individual performance differences. The sequence of rotation task trials is summarized in Fig. 4. The experimental session ended with asking participants for demographic data, consciously accessible considerations contributing to voluntary choice beyond individual performance differences (e.g., “With which strategy were your answers more accurate?”; for details, see Online Supplemental Materials (OSM)), and the need for cognition scale (Cacioppo & Petty, 1982). Specifically, we included the German implementation (Bless et al., 1994) of the need for cognition scale to explore its ability to explain how participants decide between mental and manual rotation.

#### Analyses

##### Data cleaning

Trials with no answers after 12 s (1.0% of all non-practice trials) were coded as incorrectly answered trials with a 12-s RT. Trials with an extreme RT (outside of 4 SD around individual mean) were excluded from data analysis (0.4% of remaining trials).

##### Hypotheses testing

For investigating RT and accuracy differences after completed calibration (*H1*), the *bimodality coefficient* (BC; SAS Institute Inc., 1990; see also Pfister et al., 2013) as well as *Hartigan’s dip statistic* (HDS; Hartigan & Hartigan, 1985) were computed for the individual RT (*H1-1*) and accuracy (*H1-2*) differences between Block 7 (internal) and Block 8 (extended). A combined use of both methods has been shown to validly detect bimodal distributions (Freeman & Dale, 2013). Here, we therefore use a BC of below BC_critical =_ 0.55 in combination with a nonsignificant HDS (*p* > .05) as indicator of a unimodal distribution for both *H1-1* and *H1-2*.

For investigating whether participants perform equally well with internal and extended strategies (*H2*), two dependent *t*-tests with strategy (internal, extended) as independent and RT or accuracy, respectively, as dependent variables were used. Data from forced-choice trials after calibration, i.e., from Calibration Blocks 7 and 8, were used for these analyses. Nonsignificant *t*-tests (*p* > .05) would suggest similar performance. Even though they are poorly suited for testing similarity rather than difference, *t*-tests were used because they are popular and widely understood. Therefore, the *t*-tests were complemented by Bayesian estimations (Kruschke, 2013). We aimed for a calibration accuracy that should prohibit that performance differences between mental and manual rotation are overly meaningful. Here, we defined meaningfulness in terms of the region of practical equivalence (ROPE; e.g., Kruschke, 2013). For RT differences, we decided on a ROPE from -200 to +200 ms. For accuracy, we decided on a ROPE from -2% to +2%. ROPEs were chosen to balance the need for sufficiently similar performance between mental and manual rotation and reasonable sample size. Please note that mental and manual performance profiles with HDIs falling within these ROPEs would indicate much more similar performance profiles than reported for naive non-calibrated performance profiles of both internal and extended strategies in a highly similar paradigm (especially with respect to accuracy; mean difference of ~ 6% in Weis & Wiese, 2019b).

To test whether participants continued to perseverate on both internal and extended strategies (*H3-1*), BC and HDS were used again to detect a possible bimodal distribution – this time regarding *extended strategy use*. Extended strategy use resembles the proportion of trials in the choice block during which participants engaged in manual rotation, i.e., pressed one of the arrow keys that afforded working stimulus rotation. A BC above BC_critical =_ 0.55 in combination with a significant (*p* < .05) HDS would indicate that extended strategy use has more than one mode. If visual inspection confirms the existence of one mode below an extended strategy use of .5 and one above .5, *H3-1* is confirmed. Previous studies suggest that participants are sometimes biased towards extended strategy use (e.g.; Gilbert et al., 2019; Virgo et al., 2017). Therefore, even if *H3-1* was rejected, participants could still have exhibited perseveration behavior and exclusively perseverated on the extended strategy. To test this hypothesis in *H3-2*, a one-sample *t*-test with extended strategy use as dependent variable was employed to test whether participants used manual rotation in more than half the trials (*μ* = .5). *H3-2* is only tested if *H3-1* was rejected.

#### Open science

Data, stimulus materials, and analytic code are available in an online repository [https://osf.io/vk9be/]. The experiment was preregistered [https://osf.io/4pzh7].

### Results

#### Descriptives

**Fig. 5:**
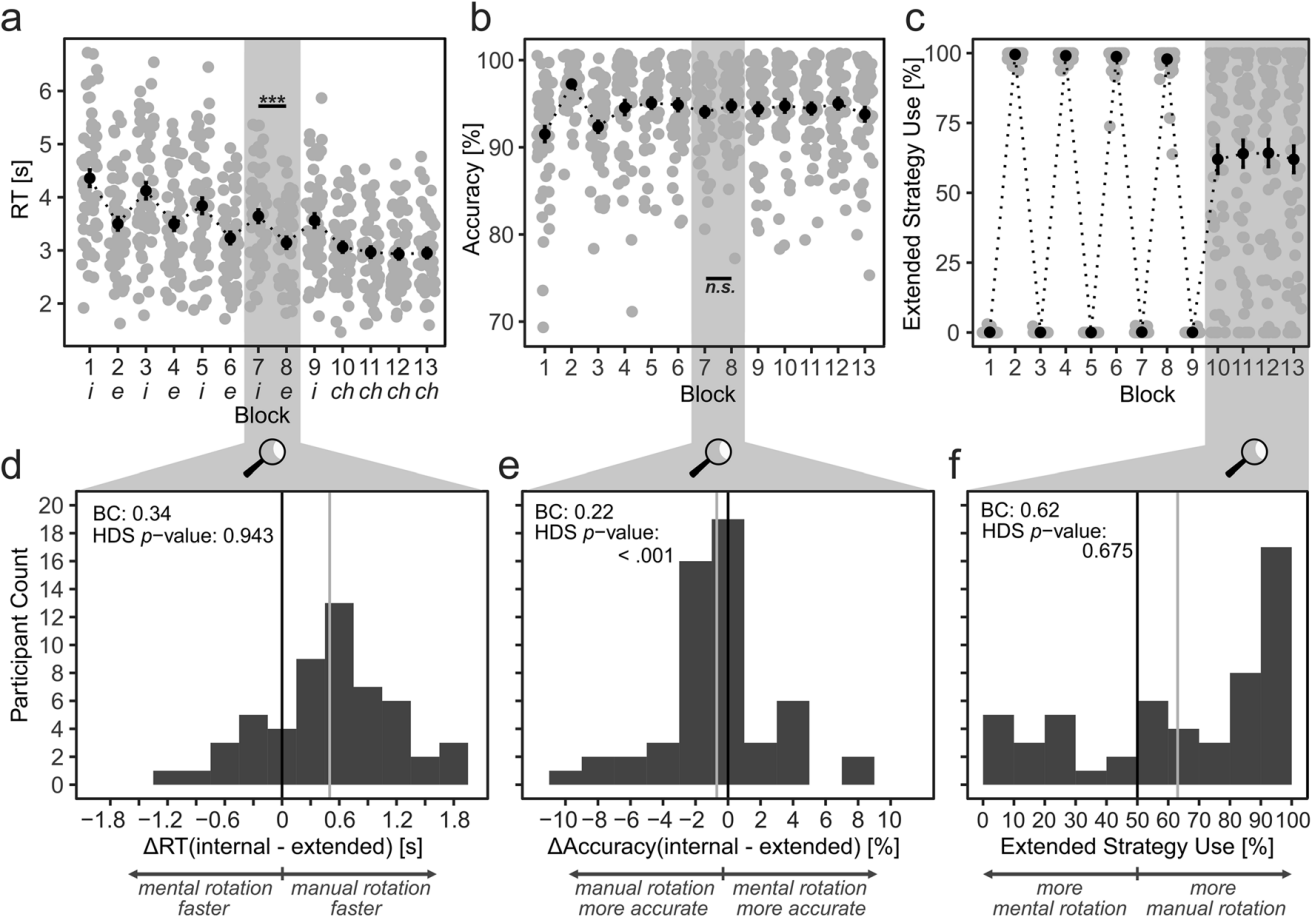
Results of Experiment 1. *Note*. The upper half depicts performance (**a, b**) and how frequently participants used manual rotation (**c**) over time. Note that during Blocks 1–9, participants were instructed to exclusively use either an internal cognitive strategy (mental rotation) or an extended cognitive strategy (manual rotation). During blocks 10–13, participants were able to freely choose between both cognitive strategies. To test calibration quality, i.e., whether internal and extended strategies have similar performances at the end of calibration, Blocks 7 and 8 were compared (**d, e**; H2). To test for perseveration, the distribution of individual extended strategy use was investigated (**f**; H3). In a–c, gray dots represent individual means in the respective block; black dots represent grand means. In d–f, black lines represent expected mean if internal and extended strategies had identical performance (**d, e**), and extended strategy use proportion was random (**f**); gray lines represent actual means. *i*: internal block, *e*: extended block, *ch*: free-choice block, *BC*: bimodality coefficient, *HDS*: Hartigan’s dip statistic, *** : *p* < .001, *n.s.* : *p* > .1

To provide an overview over the present data, we provide performance and extended strategy use means over the course of the experiment (Figs. 5a-c). Reactions were slow but not out of proportion (for the present paradigm, RTs around 2.5 s had already been reported for accuracy-oriented participants previously; Weis & Wiese, 2019b) and got faster over time (Fig. 5a). Accuracy stagnated at a high level; (Fig. 5b). Participants followed instructions, i.e., they used the different strategies as instructed during Calibration Blocks 1–9, and participants frequently but not exclusively used the extended strategy during Free Choice Blocks 10–13 (Fig. 5c). Also, as indicated by the final angles of the working stimuli during manual rotation, participants mostly followed instructions and rotated clockwise; compare Fig. S1 (OSM). Accuracy-related calibration resulted in, on average, 8.9% of unreliable trials in the choice block, which were, however, not related to extended strategy use (see Fig. S6 (OSM) for details). Overall, data quality seems reasonable.

#### Hypotheses testing

H1-1 and H1-2 were confirmed (Figs. 5d and e): After calibration,^7^ performance differences between internal and extended strategies were not bimodally distributed. Rejection of bimodality was indicated by BCs of .35 and .24 for RT and accuracy data, respectively. Rejection was also indicated by a nonsignificant HDS for RT data (*D* = .04, *p* = .68). Rejection of bimodality was not indicated by the significant HDS for accuracy data (*D* = .14, *p* < .001), which we, however, hesitate to further interpret because the HDS is in that case a less than optimal measure due to the discrete nature of accuracy differences that were multiples of 1/48. Visual inspection of the data clearly suggests a unimodal nature of the data (Fig. 5e). Confirmation of H1 excludes the possibility of large sample subgroups that exhibit a pronounced proficiency in using either the internal or the extended strategy even after calibration.

H2-1 was rejected and H2-2 confirmed (Fig. 5d and e): After calibration, performances of internal and extended strategies were comparable only with respect to accuracy but not with respect to RT. Corresponding *t*-tests indicated that performances of internal and extended strategies were similar with respect to accuracy (Δ_accuracy_ = -.005, *t*(53) = 1.00, *p* = .322) but not with respect to RT (Δ_RT_ = 511 ms, *t*(53) = 5.34, *p* < .0001). These results were mirrored by the ROPE analysis. The accuracy 95% HDI was within the ROPE of -2% to 2%: HDI_accuracy_ = -1.3% to .05%. The RT 95% HDI was completely outside the ROPE of -200 ms to 200 ms: HDI_RT_ = 322 ms to 712 ms.^8^

The preconditions for testing H3 were thus only partially met; performances of internal and extended strategies were only comparable with respect to accuracy, not with respect to RT. Keeping that in mind, the data seems ambiguous regarding the number of nodes (Fig. 5f). Although a BC of .62 suggests a bimodal distribution, the HDS suggests otherwise (*D* = .04, *p* = .68); partial confirmation of H3-1. Whereas participants used the extended strategy in more than 50% of choice trials (*M* = .63, *t*(53) = 2.88, *p* = .006, *μ* = .5; suggesting confirmation of H3-2), it is unclear whether this preference is tied to performance differences or to performance-independent perseveration tendencies. We investigated this issue further using an explorative analysis (see Explorative analyses) and ultimately a follow-up experiment (Experiment 2).

#### Exploratory analyses

To further explore the influence of performance differences on extended strategy use, two linear regressions were performed with extended strategy use as DV and accuracy or RT differences between extended and internal strategy as IVs, respectively. The model with accuracy differences as predictor did not explain extended strategy use, *F*(1, 52) = .45, *p* = .504, *R*^2^_adj_ = -.01. However, the model with RT differences as predictor did explain extended strategy use to a moderate degree, *F*(1, 52) = 12.51, *p* < .001, *R*^2^_adj_ = .18. Specifically, extended strategy use equaled 0.52 + 0.22*RT_Δ(internal – extended)_. In other words, a participant who was 1 s slower when using the internal in comparison to the extended strategy was expected to rotate 22 percentage points more frequently with the manual strategy during the free-choice blocks. These results further confirm the validity of the accuracy calibration. They, however, also pinpoint the relevance of the failed RT calibration for cognitive strategy choice.

We also explored the influence of angle (compare Fig. 3b) on extended strategy use. The employed ANOVA revealed that a higher angle is associated with an increased extended strategy use, *F*(1.4, 72.2) = 19.7, *p* < .001, η_G_2 = .01, *M*_60**°**_ = 57.8%, *M*_120**°**_ = 64.4%,* M*_180**°**_ = 67.2%. Yet, since rotation angle varied randomly throughout both calibration and choice block, it is unable to account for the target behavior of the present work, which is strong strategy preference as indicated by extended strategy use below 10% or above 90%.

### Discussion

#### Why did response time (RT) calibration fail?

Calibration consisted of two parts: Adjustments of manual rotation speed to adjust extended strategy RT and adjustments of manual rotation failures to adjust extended strategy accuracy. Whereas accuracy adjustments worked out, leading to comparable accuracy of both internal and extended strategies, RT adjustments did not. We ran two linear models – one for the internal strategy (Block 7) and one for the extended strategy (Block 8) – with angle as IV and RT as DV. Results indicated that slope adjustments were not sufficient for calibrating RT since the 511-ms difference between internal and extended strategies was accounted for by the intercept, and thus independent of the slope. This shortcoming was remediated in Experiment 2.

### Conclusion

Several participants heavily relied on the extended strategy (17 out of 54 participants, ≥ 90%) and some participants heavily relied on the internal strategy (5 out of 54 participants, ≥ 90%). Thus, 22 out of 54 or 41% of participants had strong preferences for one of both strategies. This pattern suggests frequent perseveration on the extended strategy (compare H3-2), but not on both internal and extended strategies (compare H3-1). However, the preference for the extended strategy might well originate in RT performance differences. Thus, it is unclear whether participants would have perseverated on the extended strategy without RT differences. What is clear, however, is that participants perseverated even without pronounced accuracy differences between extended and internal strategies. It could be speculated that accuracy calibration decreased perseveration in the present experiment compared to what has been reported in previous studies (i.e., 41% of participants used one strategy – internal or extended – in at least 90% of trials in the present experiment vs. on average 54% in previous studies; compare Fig. 1). However, when only comparing the present experiment with results from the same paradigm, differences vanish (i.e., 41% vs. 43%; compare Fig. 1g). However, in that study, participants showed the opposite perseveration pattern to that in the present experiment: the internal strategy was preferred over the extended strategy (Fig. 1g; Weis & Kunde, 2022). This opposite perseveration pattern might have been due to RT performance differences: While using the internal strategy was slower than using the extended strategy use in the present experiment, using the internal strategy was faster than using the extended strategy in the former study,^9^ which supports the idea that RT differences could be the driving force behind perseveration. To validate this idea, we decided to fix the RT calibration procedure and investigate the impact of similar RT differences on extended strategy use in Experiment 2.

## Experiment 2

For Experiment 2, procedure and task stayed identical with one major exception: we now included a *lockout window* for the extended strategy that delayed on-screen stimulus rotation after a rotation key was initially pressed each trial by 511 ms. In other words, when participants first pressed a rotation key, they needed to keep pressing for 511 ms until the working stimulus started rotating. This procedure was supposed to eliminate the RT differences after calibration in Experiment 1 (compare H1-2). An alternative would have been to adjust for threshold on top of adjusting for slope. However, given the already lengthy calibration process, we decided for the lockout window instead. Additionally, we fixed a minor technical issue in which slope calibration updates were occasionally miscalculated for a minority of participants^10^ and decreased the upper manual rotation speed limit from 1000°/s to 750°/s.

### Methods

#### Participants

Mirroring Experiment 1 and following the identical preregistered procedure,^11^ data collection stopped as soon as 54 participants (age mean 25.2 years, range 19–39 years; 45 female, nine male) fulfilled the inclusion criteria. Twelve participants were excluded based on the same criteria used in Experiment 1: Eight participants did not rotate in at least 85% of all extended blocks combined (i.e., blocks 2, 4, 6, and 8). One participant performed outside of 2.5 standard deviations around the group RT mean, and three participants performed below 80% accuracy in all internal blocks (i.e., blocks 1, 3, 5, 7, and 9).

For full disclosure, we again want to note that we underestimated standard deviations in the power analysis underlying the preregistered target sample size. Actual standard deviations in Experiment 2 were 591 ms and 4.0% instead of the estimated 300 ms and 2.0%. Re-running power estimations with updated standard deviations resulted in a power of the accuracy-based ROPE analysis of about 0.85 and of the RT-based ROPE analysis of only about 0.3. If willing to broaden the RT ROPE from -200 to 200 ms to -350 to 350 ms, power would increase to about 0.99. We decided to stick with the preregistered sample size while keeping possible power issues in mind for the interpretation of results.

#### Apparatus and stimuli

The same setup and stimuli as in Experiment 1 were employed.

#### Analyses

Trials with no answers after 12 s (0.5% of all non-practice trials) were coded as incorrectly answered trials with a 12-s RT. Trials with an extreme RT (outside of 4 SD around individual mean) were excluded from data analysis (0.3% of all trials). The same analyses as in Experiment 1 were employed.

### Results

#### Descriptives

**Fig. 6:**
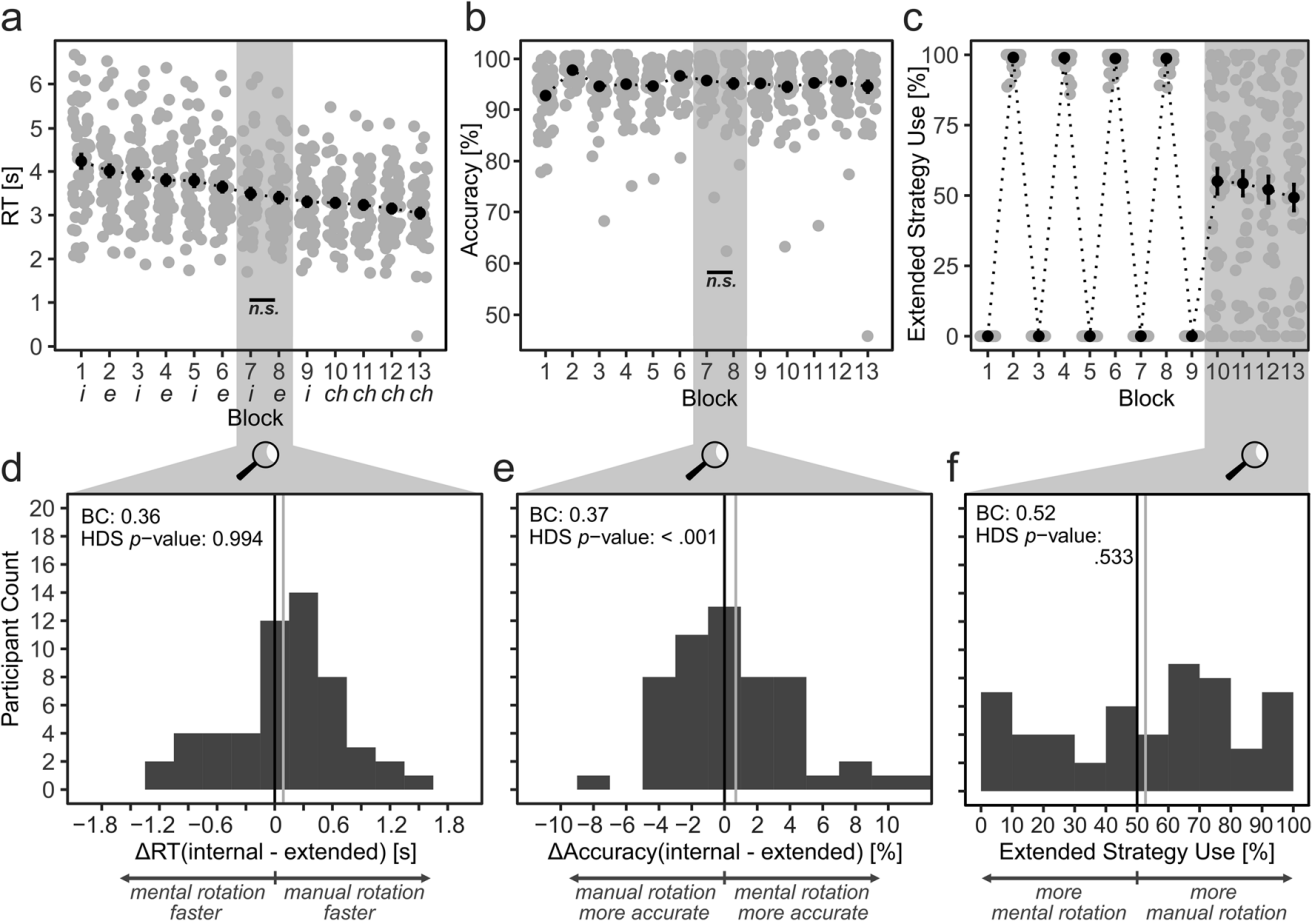
Results of Experiment 2. *Note*. The upper half depicts performance (**a, b**) and how frequently participants used manual rotation (**c**) over time. To test calibration quality, i.e., whether internal and extended cognitive strategies have similar performance at the end of calibration, Blocks 7 and 8 were compared (**d, e**; H2). To test perseveration, the distribution of individual extended strategy use proportions was investigated (**f**; H3). In a-c, gray dots represent individual means in the respective block; black dots represent grand means. In d-f, black lines represent expected mean if internal and extended strategies had identical performances (**d, e**), and strategy choice was random (**f**); gray lines represent actual means. *i*: Internal block, *e*: extended block, *ch*: free-choice block, *BC*: bimodality coefficient, *HDS*: Hartigan’s dip statistic, *n.s.* : *p* > .2

To provide an overview over the present data and allow comparisons with Experiment 1, we again provide performance and extended strategy use means over the course of the experiment (Fig. 6a-c). As in Experiment 1, reactions were slow but not out of proportion and got faster over time (Fig. 6a). Importantly, and in contrast to Experiment 1, descriptives now already indicate similar RTs for internal and extended blocks (Fig. 6a). As in Experiment 1, accuracy stagnated at a high level (Fig. 6b). Also, again, participants followed instructions, i.e., they used the instructed strategies during Calibration Blocks 1–9, and participants frequently but not exclusively used the extended strategy during Free Choice Blocks 10–13 (Fig. 6c). As in Experiment 1, participants mostly followed instructions and rotated clockwise (compare Fig. S2 (OSM)). Accuracy-related calibration resulted in, on average, 9.9% of unreliable trials in the choice block, which were, however, not related to extended strategy use (see Fig. S6 (OSM) for details). Overall, data quality seems reasonable.

#### Hypotheses testing

As in Experiment 1, H1-1 and H1-2 were confirmed (Fig. 6d and e). Rejection of bimodality was indicated by BCs of .36 and .37 for RT and accuracy data, respectively. Rejection was also indicated by a nonsignificant HDS for RT data (*D* = .03, *p* = .99). It was not indicated by the significant HDS for accuracy data (*D* = .104, *p* < .001), which we again do not further interpret as this statistical significance is an artefact stemming from the discrete nature of accuracy differences, which were multiples of 1/48; visual inspection confirmed the unimodal nature of accuracy differences (Fig. 6e). Again, this confirmation of H1 excludes the possibility of large sample subgroups that exhibit a pronounced proficiency in using either the internal or the extended strategy even after calibration.

H2-1 was mostly and H2-2 fully confirmed (Fig. 6d and e): Performances of internal and extended strategies were comparable with respect to both accuracy and RT. Corresponding *t*-tests suggest that performances of both strategies were similar with respect to accuracy (Δ_accuracy_ = .006, *t*(53) = 1.06, *p* = .294) and RT (Δ_RT_ = 89 ms, *t*(53) = 1.10, *p* = .275). These results were mostly mirrored by the ROPE analysis. The accuracy 95% HDI was within the ROPE of -2% to 2%: HDI_accuracy_ = -0.7% to 1.5%. However, the RT 95% HDI was only partly within the ROPE of -200 ms to 200 ms: HDI_RT_ = -70 ms to 256 ms. Strictly speaking, RTs for extended and internal strategies can thus not be considered similar using the preregistered ROPE criterion. Nevertheless, for the present purposes, we argue that the found HDI is still close enough to similarity. First, the assumed SD for RT differences was substantially lower than the actual SD (300 ms vs. 591 ms in the Experiment 2), which renders the preregistered ROPE from -200 to 200 ms more conservative than initially expected. Second, we provide complementary analyses in the Exploratory analyses section that show a relationship between RT and extended strategy use for Experiment 1 but not for Experiment 2. To us, this decrease from *r*_Experiment 1_ = .45 to *r*_Experiment 2_ = -.02 signified a sufficient increase in RT calibration quality to warrant interpretation of the H3-related analyses in Experiment 2.

Thus, in contrast to Experiment 1, the preconditions for testing H3 were met: internal and extended strategy performances were comparable with respect to both accuracy and RT. H3-1 was rejected. With proper calibration in place, both BC (.52) and HDS (*D* = .05, *p* = .53) offer no evidence for substantial perseveration on both internal and extended strategies. H3-2 was also rejected. Data did not suggest extended strategy use in more than 50% of choice trials (*M* = .53, *t*(53) = .64, *p* = .525, *μ* = .5). In sum, H3 was rejected, and we conclude that performance calibration can abolish perseveration.

#### Exploratory analyses

To further investigate whether adding RT-based calibration in Experiment 2 decreased perseveration on the extended strategy over only accuracy-based calibration in Experiment 1, we compared extended strategy use between both experiments. A one-sided independent *t*-test suggests that RT-based calibration did reduce perseveration; *t*(106) = 1.67, *p* = .048.

As in Experiment 1, we explored the influence of angle (compare Fig. 3b) on extended strategy use. Again, the employed ANOVA revealed that a higher angle is associated with an increased extended strategy use, *F*(1.5, 81.3) = 39.5, *p* < .001, η_G_2 = .03, *M*_60**°**_ = 45.3%, *M*_120**°**_ = 54.1%,* M*_180**°**_ = 58.8%, in line with earlier findings (e.g., Weis & Wiese, 2019a). As noted before, rotation angle varied randomly from trial to trial and can thus not account for the target behavior of the present work, which is strong strategy preference as indicated by extended strategy use below 10% or above 90%.

##### Does performance still affect extended strategy use proportion?

To double-check whether there is residual influence of performance differences on extended strategy use despite our calibration procedure, as in Experiment 1, two linear regressions were performed with extended strategy use as DV and accuracy or RT differences between extended and internal strategies as IVs, respectively. As in Experiment 1, the model with accuracy differences as predictor did not explain extended strategy use, *F*(1, 52) = 3.1, *p* = .084^12^, *R*^2^_adj_ = .04. Moreover, unlike for Experiment 1 and after successful RT calibration, the model with RT differences as predictor now also was not able to explain extended strategy use, *F*(1, 52) = .31, *p* = .908, *R*^2^_adj_ = -.02. The difference in predictive value of RT differences for extended strategy use is also reflected by significantly different correlations between RT and extended strategy use (*r*_Experiment 1_ (*df* = 52) = .45, *r*_Experiment 2_ (*df* = 52) = -.02, *z* = 2.50, *p* = .014; calculated with the function cocor.indep.groups of R’s cocor package version 1.1.4). Taken together, these analyses suggest a reduced influence of performance on cognitive strategy choice after calibration.

However, this finding does not necessarily mean that the choice process is independent of perceived performance. That performance was indeed relevant for our participants is strongly suggested by analyses of the open answers to why participants decided to use one strategy more frequently than the other. In Experiment 1, 44% of participants and in Experiment 2, 65% of participants mentioned performance as a reason (Fig. S3 (OSM)).

## Is there something special about the people who still perseverated?

For extended strategy uses, our preregistered analyses found no substantial deviation from a unimodal distribution (Fig. 6f). Nevertheless, seven participants used the internal strategy in more than 90% of trials and seven participants used the extended strategy in more than 90% of trials. To explore the underlying reasons, we took a look at metacognitive reflections on the one hand and performance differences on the other hand.

Regarding metacognitive reflections, we asked people why they preferred the strategy they used more frequently in the choice block. However, we found no obvious and unexpected differences between different participants who perseverated on internal or extended strategy, respectively (Fig. S3 (OSM)). Most prominently, around two-thirds of participants who perseverated on either strategy mentioned performance as the reason for choosing their preferred strategy in the choice block.

When looking at actual performance data, however, participants who perseverated in Experiment 2 did not exhibit particularly pronounced performance differences between the two strategies (Fig. S4). The only noteworthy descriptive difference might be that participants who perseverated on the internal strategy did indeed exhibit higher speed for mental rotation at the end of calibration; ΔRT_mental Block 7 – manual Block 8_ = -307 ms. But such differences were not uncommon for participants who did not perseverate, either (Fig. S4 (OSM)). Potential relevance of the 307-ms difference is reflected by a positive but statistically non-significant correlation between RT_internal_ – RT_extended_ and extended strategy use in the choice block; *r* = 0.31, *t*(12) = 1.15, *p* = 0.274 (calculated for the 14 participants who perseverated only). Another reason for perseveration is more likely: participants’ metacognitive estimations of their RT differences (RT_internal_ – RT_extended_) did predict – more precisely: postdict – extended strategy use more convincingly than actual RT differences. After conclusion of the main experiment, we asked participants “With which strategy were you able to respond faster?” and answers were coded on a Likert scale ranging from 0 (“Inner Eye”)^13^ to 4 (“Keyboard”)^14^. The more participants *thought* that the extended strategy (“Keyboard”) was faster, the more they used it; *r* = 0.67, *t*(12) = 3.13, *p* = .009. The same analysis with ratings for accuracy rather than speed was inconclusive; *p* = .351. Lastly, the need for cognition was not associated with extended strategy use in the 14 perseverating individuals. Such an association would have indicated that a performer’s cognitive style, for example, striving to solve things with their own cognitive abilities, could determine perseveration. Interestingly, the non-significant correlation suggested that a higher need for cognition is associated with higher *extended* rather than internal strategy use; *r* = 0.38, *t*(12) = 1.43, *p* = .179 (the correlation was also non-significant for the whole sample; *r* = 0.21, *t*(52) = 1.51, *p* = .137) .

To conclude, it is likely that more than one reason led to perseveration in these 14 participants. In this respect our study adds to the growing body of research on the reasons for what has been called *psychological momentum*, hence a general tendency of mental states to persist and return to mind (Honey et al., 2023). The most prominent reason for the present instance of such momentum might be metacognitive evaluations of speed differences between both strategies, though we want to remind the reader of the highly exploratory nature of this finding. Other less prominent reasons that our participants mentioned at the end of the study session in an open answer format included fatigue, effort avoidance, avoiding unreliable extended trials, the possibility to double-check answers when using the extended strategy, and mental challenge seeking (Fig. S3 (OSM)).

Results indicate that performance calibration was successful in Experiment 2. In Experiment 1, preregistered analyses suggested a large degree of perseveration. In Experiment 2 however, identical analyses delivered little evidence for perseveration: extended strategy use was neither above chance level nor distributed bimodally.

## General discussion

### Likely reasons for perseveration: Actual and believed performance

The present results suggest that strong preferences for a specific cognitive strategy – i.e., perseveration – as observed in previous studies (Fig. 1) or similarly in Experiment 1 of the present study (Fig. 5f) are to a large degree due to performance differences between available cognitive strategies. With diminishing performance differences between the two strategies available in the present study, perseveration ceased to dominate the histogram in Experiment 2 (Fig. 6f). Interestingly, some individuals still exhibited predominant use of either mental or manual rotation. Exploratory analyses offer a first hint that inaccurate metacognitive speed estimations might have contributed to perseveration in these individuals. In sum, the present results thus suggest that strong preferences for a specific cognitive strategy throughout a whole experiment are caused by the same *usual suspects* that are in play when deciding between different cognitive strategies without perseveration: actual performance (e.g., Gilbert, 2015; Gray et al., 2006; Walsh & Anderson, 2009; Weis & Wiese, 2019b) and possibly also metacognitive performance estimates (e.g., Gilbert, 2015; Risko & Dunn, 2015; Risko & Gilbert, 2016; Touron, 2015).

### Unlikely reasons for perseveration

This substantial influence of performance considerations on cognitive strategy perseveration came as a surprise to us. In fact, the influence seems so substantial that there is little space for other considerations that initially seemed probable.

First, we deemed it reasonable that participants particularly avoid mental effort that is conceivably higher with internal strategy use (e.g., Sachdeva & Gilbert, 2020). However, there seems little space for this cause in the present data: perseveration on the extended strategy was rare after performance calibration in Experiment 2, and only one of the seven participants who perseverated on the extended strategy explicitly mentioned effort avoidance as the underlying reason. Descriptively, effort avoidance might be as rare a reason for perseveration as its counterpart, mental challenge seeking (Inzlicht et al., 2018), which was mentioned by one of the five participants who perseverated on the internal strategy in Experiment 1. In the same line, it would have been conceivable if individual differences in cognitive style as captured by the need for cognition scale explained some perseveration on internal (vs. extended) strategies, which it did not.

Second, participants might perseverate to avoid possible costs of deciding between two strategies, as compared to working with one strategy only. Mixing and switching costs have been associated with cognitive effort and demand on executive functions (Kool et al., 2010) and were found both for voluntary task switches (Arrington & Logan, 2004; Kiesel et al., 2010) and for voluntary cognitive strategy switches (Weis & Kunde, 2022). The generally low level of perseveration after calibration makes choice cost avoidance an unlikely reason for perseveration, despite the fact that substantial RT-based switching costs were reported for the same paradigm as employed in the present study (Weis & Kunde, 2022). Additionally, we found no evidence for choice cost avoidance in the open answers. This all was observed despite the fact that participants who frequently used both strategies exhibited abundant switching (see Fig. S5a (OSM) for more details). Thus, participants might have accepted the effort as well as moderate RT costs (~100–200 ms; Weis & Kunde, 2022; also see Figs. S5b and S5c (OSM) for an exploration of switch costs in the present study) associated with strategy switches either because they did not detect switch costs, or switched to keep the task environment sufficiently stimulating (also see section *Limitations and future research*), or counteracted switching costs with stimulus-specific strategy choices like preferring the extended strategy for higher angles. Taken together, we cannot exclude that switch costs play some role for perseveration in the present paradigm (see also Weis & Kunde, 2022). However, the abundant switching that was present after performance calibration suggests that strategy-specific performances were ultimately more important.

Third, we deemed it likely that perseveration is partially caused by participants who opt out of performance monitoring or subsequent metacognitive use of the monitored information. Instead, we anticipated that participants might decide early on for one “good enough” strategy that allows for reasonably fast and accurate answers. Such behavior would be associated with a satisficing agent who is not willing to continuously search for the best strategy available (Simon, 1956). We found no evidence for satisficing behavior in the open answers but contrarily found behavior consistent with enduring performance monitoring. We cannot exclude that satisficing did occur, but it seems negligible in comparison to considerations more tightly linked to performance.

Fourth, theoretically, episodic retrieval could be at the roots of cognitive strategy perseveration (Frings et al., 2020). That is, responding to a stimulus in a specific way could create an episodic binding between stimulus and response (here: use of either strategy). Encountering the same or a similar stimulus a moment later would then retrieve the previous response again, and so forth. The present results indicate that while such involuntary retrieval might exist, it seems too weak, too short-lived, or too stimulus-specific to prompt enduring perseveration tendencies.

### Limitations and future research

First, we want to note that the term perseveration is defined by overt behavior in the present study and might thus not always entail perseveration on one specific cognitive strategy. For example, whenever participants exhibit overt manual rotation, they might in fact also use mental rotation in parallel. The problem of disentangling parallel strategy use from single strategy use applies to many extended cognitive strategies (as discussed in, e.g., Walsh & Anderson, 2009; Weis & Wiese, 2019a). Similarly, when participants exhibit no overt extended strategy-related behavior, several internal cognitive strategies might be used. For example, mental rotation might sometimes be skipped and replaced with other internal strategies like retrieving the correct answer from memory (e.g., Walsh & Anderson, 2009; Weis & Wiese, 2019a). We, however, refrained from asking participants about the employed strategy after each trial to prevent interrupting other relevant processing like performance monitoring. Thus, one should be mindful that what we call perseveration on an extended strategy in the present paper is defined by overt behavior and that what we call perseveration on an internal strategy might in fact encompass several internal strategies. Such considerations cannot explain our main finding, i.e., the reduction of perseveration on an extended strategy after performance calibration, but should be kept in mind when designing further studies.

Second, our participants were able to exclusively focus on one task and were hardly distracted throughout our study. If we assume that humans do not per se avoid cognitive effort, this rather unstimulating environment might have invited our participants to engage in more metacognitive control than would have happened in more stimulating environments. What else should our participants have done to avoid boredom? Recent research has shown that humans prefer some cognitive effort over doing nothing (Wu et al., 2022), and accordingly, contemplating about strategy performances might have been more tempting than doing nothing between trials. Further research could clarify whether perseveration due to performance considerations might decline in more natural situations in which humans have even more exciting things to do than comparing performances of competing cognitive strategies.

Third, the present design was not designed to replicate strategy perseveration without calibration as observed in earlier studies (Fig. 1). We deem a replication of strong perseveration in the present setup likely, given that strong perseveration has already been found with the same paradigm despite extensive prior practice with both strategies (Fig. 1g). However, strictly speaking, the present study only provides evidence for a reduction/elimination of perseveration when RT-based calibration was added in Experiment 2 on top of the accuracy-based calibration from Experiment 1. Showing that accuracy-based calibration can reduce perseveration in comparison to no calibration was out of the scope of the present investigation, but would further strengthen the present findings.

Lastly, based on the present results, we would advise looking for performance reasons first when trying to understand situations in which performers perseverate on a specific cognitive strategy, for example, when observing people who always use their smartphones to calculate. However, we acknowledge that such advice might not transfer to all situations and that further investigating possible influences of metacognitive processing, individual differences, attentional factors, or cognitive load on perseveration might prove fruitful. Here, participants were situated in a lab context and explicitly told to avoid errors as far as possible, which makes us wonder in how far our findings would generalize to everyday contexts.

## Conclusion

Why would a human repeatedly solve similar problems with the same identical strategy without employing other known strategies? The present research suggests that performance is a key factor. Most problem solvers were not reluctant to frequently switch and use both strategies if performance was comparable. Instead, problem solvers were willing to invest the cognitive upkeep necessary for employing a repertoire of multiple cognitive strategies. Our results imply that whenever one observes a predominant cognitive strategy, one should consider performance of individual strategies as a root cause rather than cognitive inflexibility or other constraints of the mind. We deem this a promising result for performers in modern environments that afford a multiplicity of strategies because it showcases a certain unwillingness to prematurely – i.e., without performance-related reasons – focus on one strategy.

### Supplementary Information

Below is the link to the electronic supplementary material.

Supplementary file1 (DOCX 1739 KB)

## Data Availability

Data, stimulus materials, and analytic code are available in an online repository [https://osf.io/vk9be/]. Experiment 1 was preregistered [https://osf.io/4pzh7].

## Footnotes

1 Only with reduced perseveration on the internal strategy if prior experience was with the extended strategy only.

2 Even though the Hartigan’s dip statistic (HDS) is non-significant as indicated in Fig. 1g, we argue that there still is unequivocal intraindividual perseveration. The question is whether participants perseverate on both internal and extended strategies as the BC suggests (see Fig. 1g) or only on the internal strategy, which is supported by a one-sample *t*-test (externalization proportion is below 0.5; *M* = 42.6, *t*(107) = 2.20, *p* = .03).

3 More precisely, for rotations of three-dimensional objects on a (two-dimensional) plane.

4 That is, across a variety of similar tasks/stimuli.

5 This criterion was set to 90% in preregistration. The criterion was lowered to avoid excessive participant exclusions (i.e., seven instead of 14 participants). Results remain similar when excluding 14 participants. However, we deem it more justified to adjust the preregistered procedure to a reasonable degree than to exclude a large proportion of participants.

6 Specifically, instructions for the choice block, translated from German, were as follows: “Excellent! You completed the first part of the main experiment. For the rest of the experiment, you can choose to solve the tasks with your inner eye (your mental imagination) or the keyboard (the left and right arrow keys). Press the [space bar] to continue.” (at the beginning of the choice block) and “Block x of 13 is about to begin. You can choose between your inner eye and the keyboard. IMPORTANT: Please avoid mistakes. Even rare mistakes can render your data unusable for us. Continue by pressing the [space bar].” between choice sub-blocks.

7 That is, at the end of the calibration block.

8 Note that the HDI would also have been outside a ROPE of -350 to 350 ms, a ROPE that would not have been underpowered; see Methods*: *Participants.

9 The reverse pattern applied for accuracy.

10 Occasional miscalculation occurred in three participants with extensive counterclockwise manual rotation. Errors were minor and participant exclusions would not have altered our conclusions drawn for Experiment 1; nevertheless, we wanted to address the issue.

11 Note that we did not hand in a separate preregistration of Experiment 2 as we deemed it unnecessary given the identical analyses.

12 Even though this result could be termed “trending,” we want to note that the trending effect would be in the unexpected direction, i.e., each 10 percentage-point accuracy advantage of the internal in comparison to extended strategy would be associated with an 18 percentage point *increase* in extended strategy use.

13 Which refers to mental rotation.

14 Which refers to manual rotation.
