# Supplementary material for: Perseveration on cognitive strategies
Source: Mem Cognit. 2023 Oct 24;52(3):459–75. doi: 10.3758/s13421-023-01475-7 (PMC11021300; doi:10.3758/s13421-023-01475-7)
Supplement: Supplementary file 1 — Supplementary file1 (DOCX 1739 KB) [file 13421_2023_1475_MOESM1_ESM.docx]

**Supplemental Materials**

**Figure S1**

*Final Angle of Manual Rotation (Extended Strategy) for Different Angle Conditions in Experiment 1*


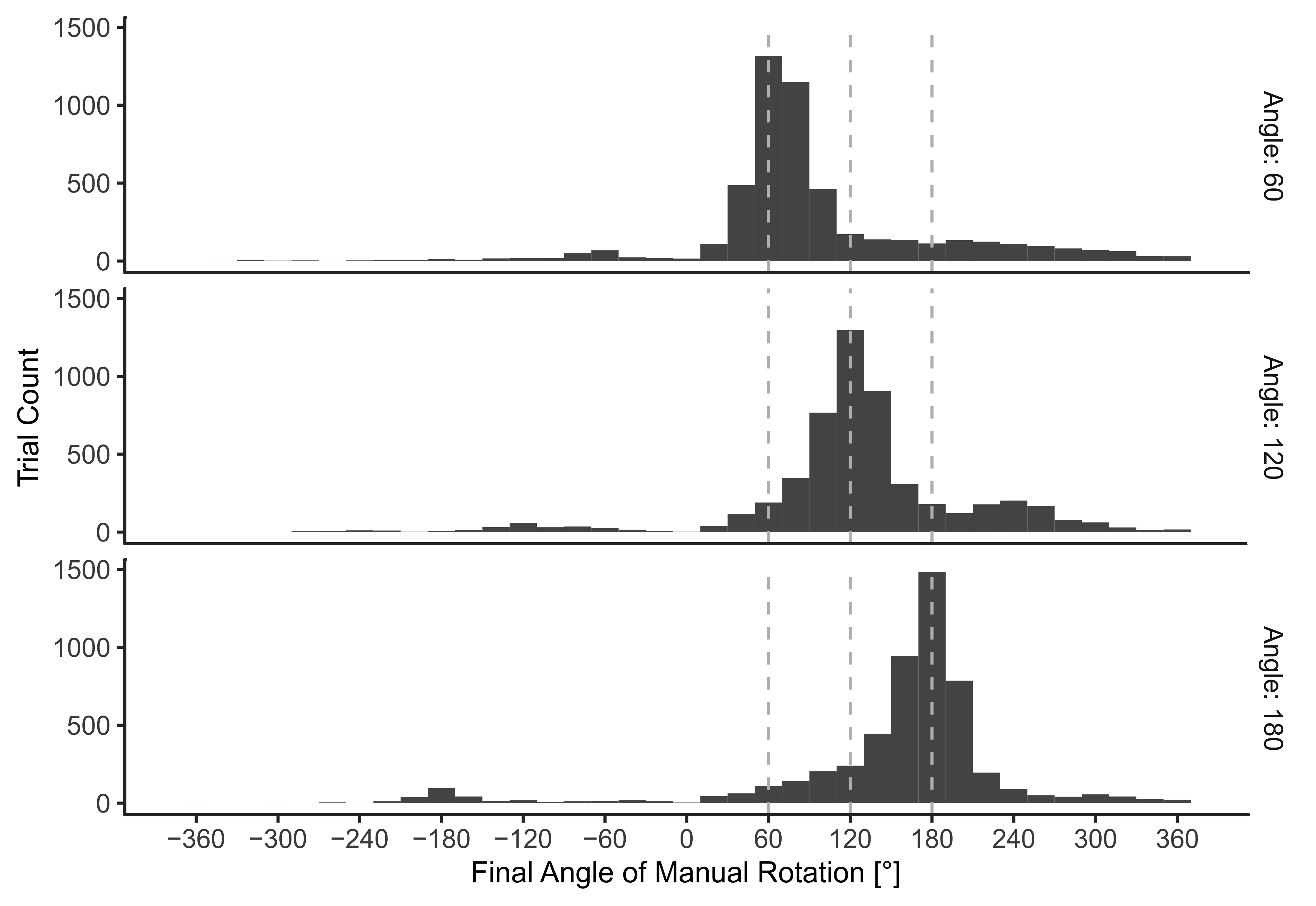


*Note*. Participants mostly followed instructions and rotated clockwise as indicated by positive *Final Angle of Manual Rotation* values. On average, participants stopped rotating the working stimulus as soon as it was aligned with the base stimulus. In other words, the median of the *Final Angle of Manual Rotation* aligns with the angle of the respective angle condition; dashed gray lines. For the present graph, all trials with more than a full 360° rotation, leading to final angles beyond -360° or 360° were omitted (551 trials or 3.7%). All other trials in which participants used manual rotation were used.

**Figure S2**

*Final Angle of Manual Rotation (Extended Strategy) for Different Angle Conditions in Experiment 2*

**
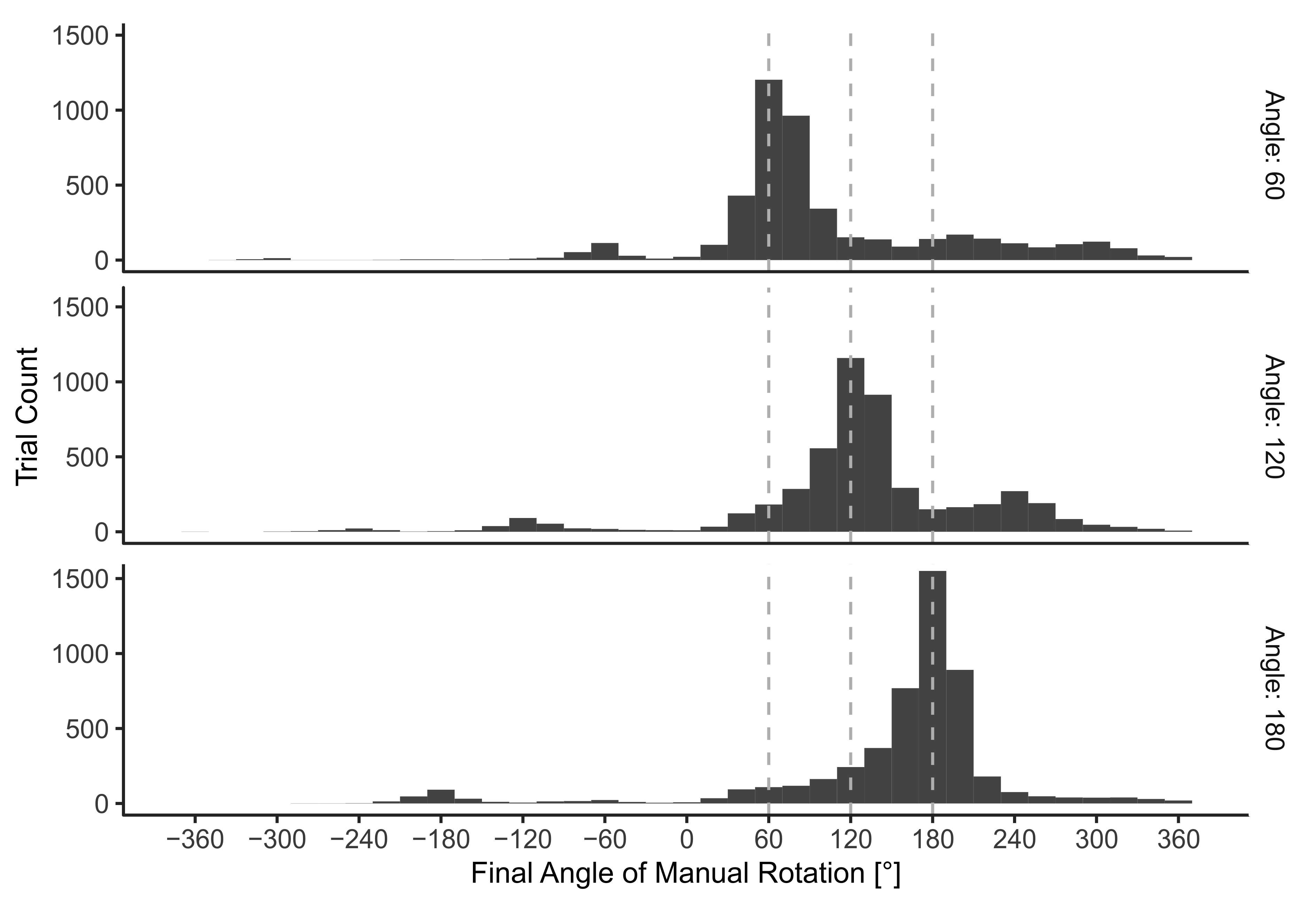
**

*Note*. As in Experiment 1, participants mostly followed instructions and rotated clockwise as indicated by positive *Final Angle of Manual Rotation* values. On average, participants stopped rotating the working stimulus as soon as it was aligned with the base stimulus. In other words, the median of the *Final Angle of Manual Rotation* aligns with the angle of the respective angle condition; dashed gray lines. For the present graph, all trials with more than a full 360° rotation, leading to final angles beyond -360° or 360° were omitted (237 trials or 1.5%). All other trials in which participants used manual rotation were used.

**Figure S3**

*Metacognitive Reasons for Cognitive Strategy Choice in the Choice Block*


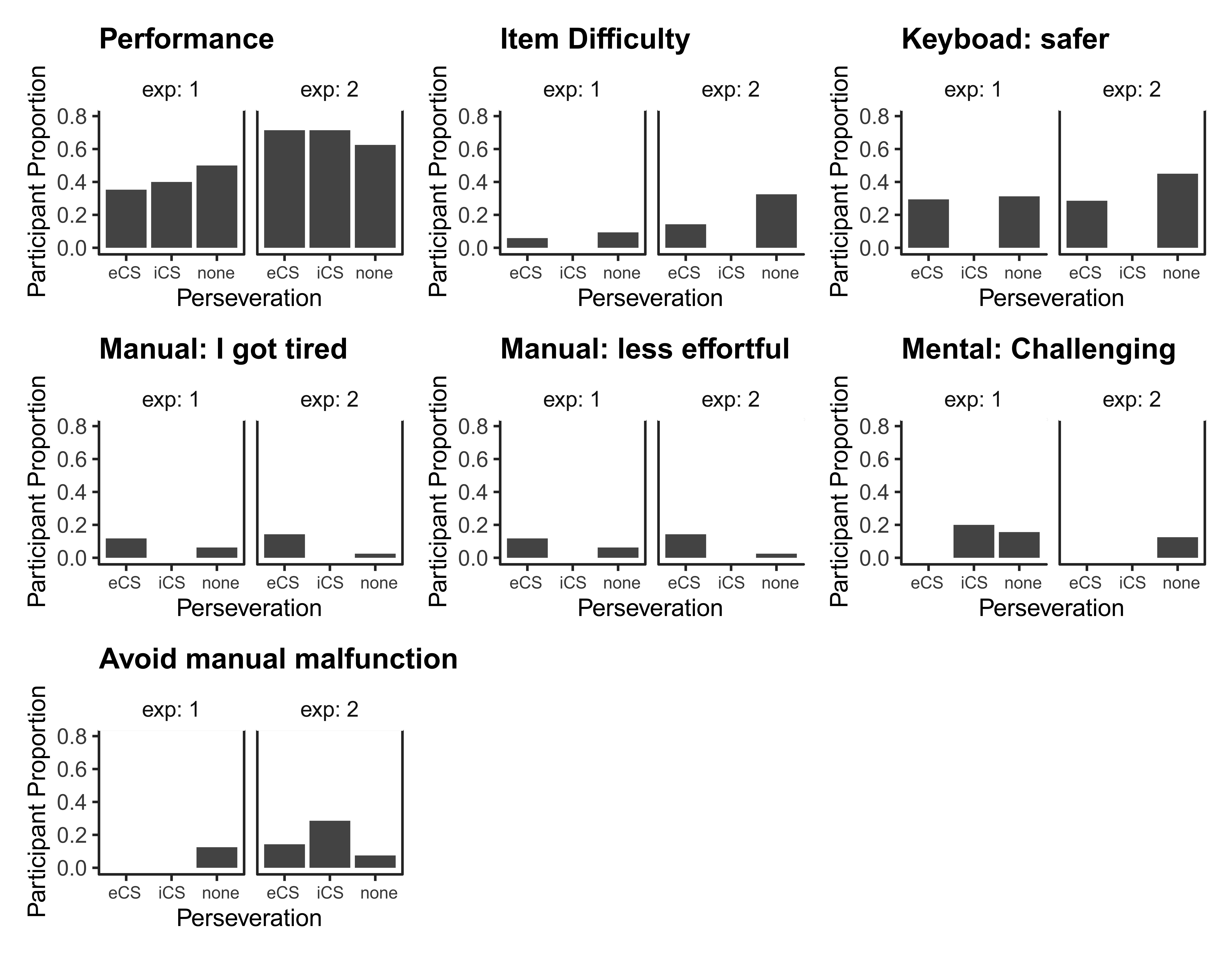


*Note*. In an open answer format, we asked people why they preferred the strategy they used more frequently in the choice block. After glancing over the answers, we coded every answer independently regarding the seven reasons listed in the figure. Please note that participant proportion is based on the respective perseveration group: for example, one of the five participants (or: 20%) who perseverated on the internal strategy in Experiment 1 did so because of enjoying the associated challenge. For an answer to be counted for a specific reason, we used German keywords that needed to be included in the open answer (performance: “error”, “faster”, “time reasons”, “success rate”, “takes longer”, “saves time”, “more accurate”, “correct answers”; item difficulty: “depending on the figure/object”, “special feature of the object”, “for some figures/objects/shapes”, “for harder/easier tasks”, “at 180 degrees”, “when objects were strongly twisted”, “more obviously dissimilar”, “for more complex tasks”; keyboard safer: “certainty”/”security” (German: “Sicherheit”), “(double-)check the answer”, “uncertain”, “insecure”, “reassure”, “verify”, “confirm”, “revise the answer” ; mental i got tired: “less focussed [..] at the end”, “less able to concentrate [...] at the end”, “hard to stay attentive [...] at the end”, “requires less concentration”; manual less effort: “effortful”, “when I was overexerted”; mental challenging: “challenge”, “wanted to do it on my own”, “more joy”, “more challenging”, “wanted to prove it to myself”; avoid manual malfunction: “objects vanished during rotation”, “loading error”, “object/image not displayed”, “errors during rotation”). exp: Experiment, eCS: extended cognitive strategy (used in more than 90% of trials), iCS: internal cognitive strategy (used in more than 90% of trials).

**Figure S4**

*Performance Differences Between Internal and Extended Cognitive Strategies*

*
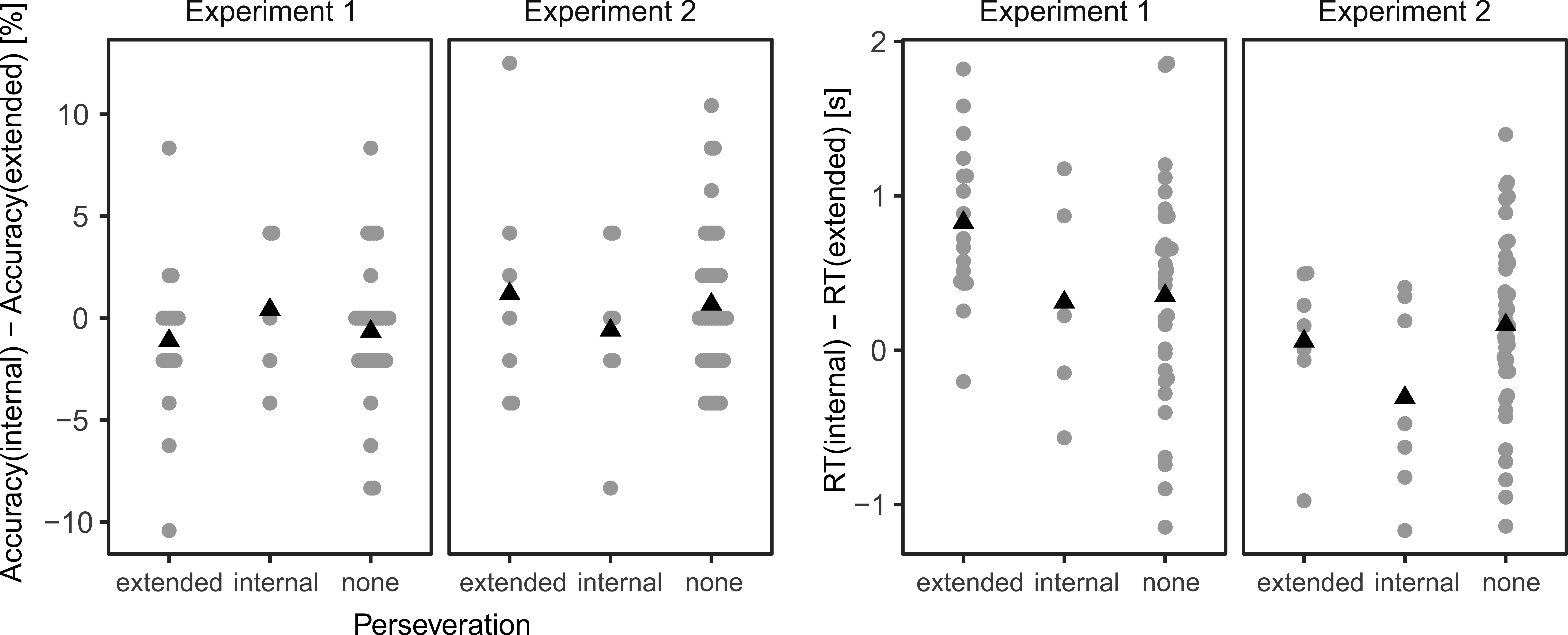
*

*Note*. Performance differences are based on performance in Blocks 7 (internal cognitive strategy) and 8 (extended cognitive strategy). Perseveration on either strategy refers to use in more than 90% of trials.

**Figure S5**

*Switch Proportions and Switch Costs*

*
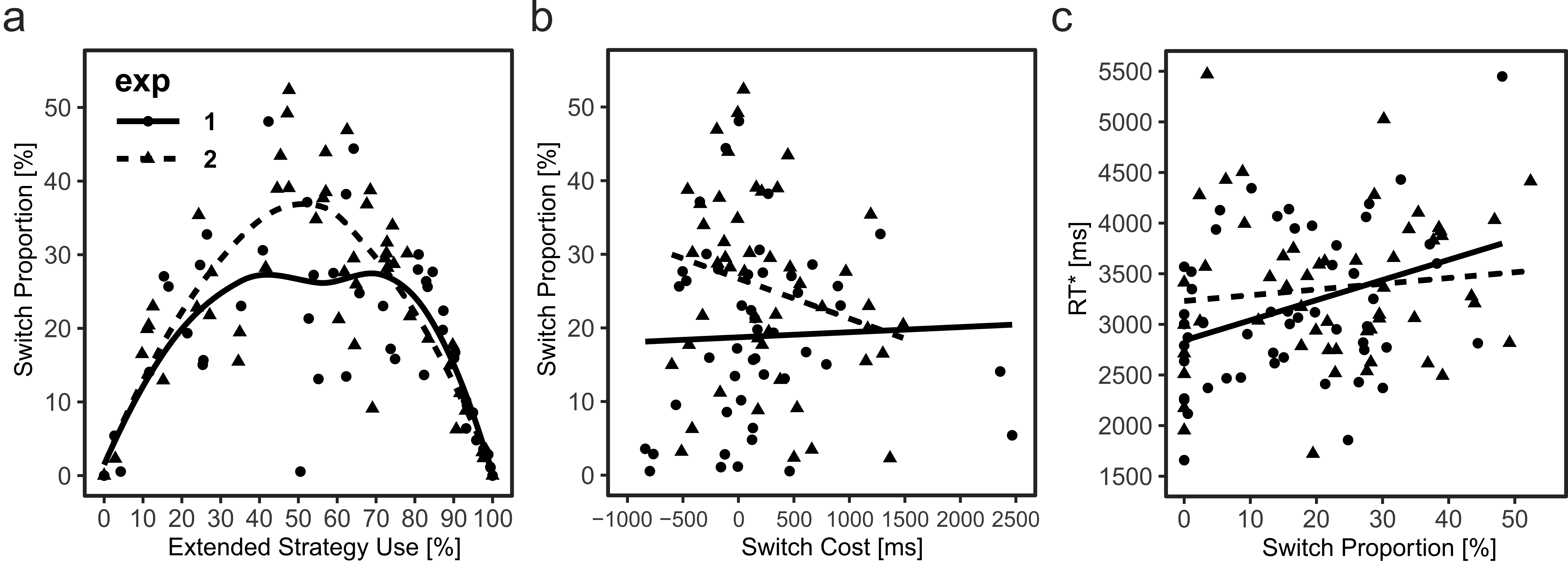
*

*Note*. Participants who used both strategies did switch frequently (a). Exploratory analyses revealed that switch costs were likely present in the current paradigm but are ambiguous whether these switch costs were related to switch proportions (b). To analyze switch costs, we used all choice trials, except for the first trial after each break, and except for trials that had been excluded due to excessive RT (outside of 4SD of individual mean). For each individual, we divided the average RT of all trials with a strategy switch by the corresponding accuracy to obtain RT*. For each individual, we then subtracted this value from the average RT* of all trials without a strategy switch. For Experiment 1, a one-sample *t*-test was ambiguous regarding the existence of switch costs; *M* = 184 ms, *t*(45) = 1.76, *p* = .086. Note that 8 individuals did not exhibit a single switch trial such that we were not able to compute switch costs and that many of the remaining participants exhibited only few switches which introduced substantial noise into the analysis. For Experiment 2, a one-sample *t*-test was more clearly indicating the existence of switch costs; *M* = 267 ms, *t*(47) = 3.14, *p* = .003. Note that 6 individuals did not exhibit a single switch trial such that we were not able to compute switch costs and that many of the remaining participants exhibited only few switches which introduced substantial noise into the analysis. In Experiment 1, switch costs were not correlated with switch proportion; *t*(44) = .39, *p* = .695, *r* = .06. In Experiment 2, the analysis appeared slightly more ambiguous regarding whether switch costs were correlated with switch proportion; *t*(46) = -1.63, *p* = .111, *r* = -.23. A positive correlation between Switch Proportion and mean RT*—i.e., RT divided by accuracy—suggests that switches were associated with costs (c); *t*(106) = 2.62, *p* = .010, *r* = .25. When looking at both experiments separately, the correlation was descriptively larger in Experiment 1 (r = .35) than in Experiment 2 (r = .11), which could however be a power issue. As for (b), switch proportions as well as RT* were computed for the choice block only. The first trial of each choice block was excluded for both measures. A switch proportion of 100% would indicate that a participant switched 47 times in each choice sub-block, which always consisted of 48 trials. Each dot/triangle refers to averages of all choice sub-blocks of a single individual. Lines refer to the loess function as implemented by ggplot2 (version 3.4.0). All in all, we conclude that—in line with the separate study focusing on switch costs (Weis & Kunde, 2022)—strategy switch costs did exist in the present study, but were only loosely correlated with perseveration tendencies.

exp = Experiment.

**Figure S6**

*Unreliable Trials in the Choice Block*

**
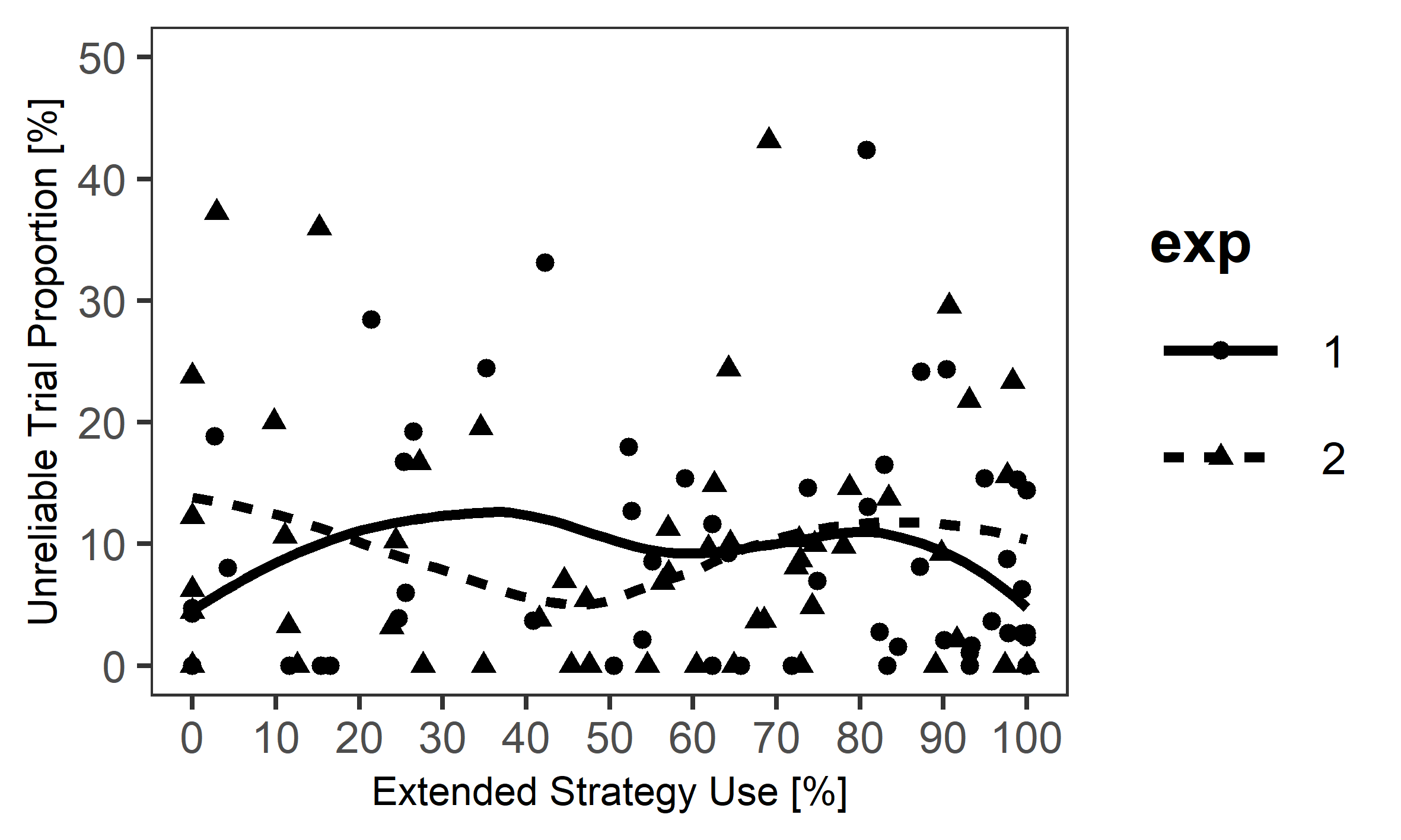
**

*Note*. How many unreliable trials were present during the choice block was determined in the calibration block. Note that a trial is defined as unreliable if extended rotation would fail *if used*, not if extended rotation did in fact fail. Each dot/triangle refers to average unreliable trial proportions during all choice trials of a single individual. Lines refer to the loess function as implemented by ggplot2 (version 3.4.0). The proportion of unreliable trials did not correlate with extended strategy use; |*r*| < .07, |*t*(52)| < .5, and *p* > .6 for each of both experiments.

exp = Experiment.

**Questions regarding CM choice (in German)**

Für welche Strategie haben Sie sich im letzten Teil des Experiments, als Sie die freie Wahl zwischen den Strategien hatten, entschieden?

I-------------------------------------------o------------------------------------------------I

ausschließlich Inneres Auge ausgeglichen ausschließlich Tastatur

Sie haben gerade ausgewählt, für welche Strategie (Inneres Auge oder Tastatur) sie sich eventuell bevorzugt entschieden haben. Warum haben Sie sich so entschieden?

Bitte tippen Sie Ihre Antwort auf der Tastatur in ein bis drei Sätzen ein. __________________________________________________________________________________________________________________________________________________________

Angenommen, Sie möchten gerne immer korrekt antworten. Welche Strategie war besser geeignet, um dabei möglichst schnell zu sein?

I-------------------------------------------o------------------------------------------------I

Inneres Auge ausgeglichen Tastatur

Mit welcher Strategie haben Sie mehr korrekte Antworten gegeben?

I-------------------------------------------o------------------------------------------------I

Inneres Auge ausgeglichen Tastatur

Mit welcher Strategie haben Sie schneller geantwortet?

I-------------------------------------------o------------------------------------------------I

Inneres Auge ausgeglichen Tastatur

Wie oft haben Sie im letzten Teil des Experiments, als Sie die freie Wahl zwischen den Strategien hatten, beide Strategien gleichzeitig (also Inneres Auge und Tastatur während einer Aufgabe) angewendet?

I---------------------------------------------o-------------------------------------------------I

nie mal so mal so immer

Wie wichtig war es Ihnen, während der Studie gute Leistungen zu erbringen?

I---------------------------------------------o-------------------------------------------------I

Nicht sehr wichtig ausgewogen Sehr wichtig
